# Genome-Wide Identification and Characterization of the *SBP* Gene Family in Passion Fruit (*Passiflora edulis* Sims)

**DOI:** 10.3390/ijms232214153

**Published:** 2022-11-16

**Authors:** Yanhui Liu, Jieyu Yuan, Dan Zhang, Kao Deng, Gaifeng Chai, Youmei Huang, Suzhuo Ma, Yuan Qin, Lulu Wang

**Affiliations:** 1 Fujian Provincial Key Laboratory of Haixia Applied Plant Systems Biology, Pingtan Institute of Science and Technology, College of Life Science, Fujian Agriculture and Forestry University, Fuzhou 350002, China; 2 Guangxi Key Laboratory of Sugarcane Biology, State Key Laboratory for Conservation and Utilization of Subtropical Agro-Bioresources, College of Agriculture, Guangxi University, Nanning 530004, China

**Keywords:** passion fruit, SBP, synteny, plant growth, stress

## Abstract

The SQUAMOSA promoter binding proteins (SBPs) gene family plays important roles in plant growth and development. The *SBP* gene family has been identified and reported in many species, but it has not been well studied in passion fruit. In this study, a total of 14 *SBP* genes were identified in passion fruit and named from *PeSBP1* to *PeSBP14* based on their chromosomal distribution. The phylogenetic tree, gene structure, conserved motifs, collinearity analysis, and expression patterns of the identified SBP members were analyzed. We classified the *PeSBP* genes into eight groups (I to VIII) according to the phylogenetic tree, gene structure, and conserved motifs. Synteny analysis found that 5 homologous gene pairs existed in *PeSBP* genes and 11 orthologous gene pairs existed between passion fruit and Arabidopsis. Synonymous nucleotide substitution analysis showed that the *PeSBP* genes were under strong negative selection. The expression pattern of *PeSBP* genes in seed, root, leaf, and flower showed that nine of the PeSBP genes displayed high expression in the leaf and the flower. The expression patterns of *PeSBP3*/*6*/*8*/*9*/*10* were further detected by qRT-PCR. In addition, differences in the expression levels occurred for each gene in the different flower organs and at the different developmental stages. There were large differences among *SBPs* based on transcriptional levels under cold, heat, salt, and osmotic stress conditions. Altogether, this study provides an overview of *SBP* genes in passion fruit and lays the foundation for further functional analysis.

## 1. Introduction

Transcriptional regulation is an important part of eukaryotic gene expression regulation, and transcription factors (TFs) play pivotal roles in plant growth and development processes [1]. TFs, including DNA binding domains, transcriptional activation domains, nuclear localization signals, and oligomerization sites [2], function by binding to corresponding gene promoters, thus activating or inhibiting the transcription of target genes [3]. TFs can be divided into different families based on their DNA binding domains [4]. In plants, 64 TF families have been identified [5].

The SQUAMOSA promoter binding (like) proteins (SBPs/SPLs) gene family is part of a class of important transcription factor families that regulates the growth and the development of green plants under stress. The SBP TFs have a conserved SBP domain containing about 79 amino acid residues that are highly conserved [6]. The SBP domain includes two typical zinc-binding sites (Cys-Cys-Cys-His, Zn1 and Cys-Cys-His-Cys, Zn2) and a highly conserved nuclear localization signal (NLS) at the C-terminal partially overlapping with the Zn2 zinc finger structural sequence [7]. The SBP protein localizes in the nucleus with the nuclear localization signal serving as a guide to regulate the transcriptional expression of downstream genes [8]. The first *SBP* gene was discovered in inflorescences of *Antirrhinum majus L.* in 1995 [9]. Since then, *SBP* genes have been found in a variety of plants, such as *Arabidopsis* [10], rice [6], soybean [11], grape [8], sorghum [12], Populus [13], pepper [14], maize [15], and tomato, amongst others [16]. SBP is a unique transcription factor in green plants which plays a very important role in a series of plant development processes. In *Arabidopsis*, the first *SBP* gene has been identified as *ATSPL3*, which regulates flowering under long photoperiod [10,17]. *ATSPL8* is a local regulator involved in the regulation of microsporogenesis and megasporogenesis [18]. *TaSPL20* and *TaSPL21* were highly expressed in the panicle of early wheat, and the ectopic expression of *TaSPL20* and *TaSPL21* in rice has similar functions in promoting panicle development [19]. Inhibition of the expression of *SlSPL13* in tomato increases the number of inflorescences on vegetative branches and side branches, reduced the number of flowers and fruits, and reduced fruit size and yield. *SlSPL13* directly binds to the promoter region of the tomato inflorescence-related *SFT* gene and positively regulates its expression, thereby controlling the development of inflorescence [20]. The high expression of *OsSPL14* in the reproductive stage of rice promotes the increase of panicle branch and thus the yield [21], and *OsSPL16* promotes the cell division of rice and changes the size and shape of grain [22]. NaCl stress can induce the expression of *BpSPL9* in birch roots and leaves, and enhanced expression of *BpSPL9* promotes reactive oxygen scavenging under stress [23]. These studies have shown that *SBP* genes play an important role in plant microsporogenesis, flowering, yield, and stress resistance. Therefore, identifying important genes in the *SBP* gene family is of great importance for identifying candidate genes for the genetic improvement of economic species.

Passion fruit (*Passiflora edulis* Sims) belongs to the Passifloraceae family and the Malpighiales order with more than 520 species in the world. It is one of the most economically important genera in the Passifloraceae family [24,25]. Passion fruit is native to subtropical regions of North and South America [26]. It is widely cultivated in tropical and subtropical regions because of its value as an easy-to-manage edible, medicinal, and ornamental crop [27]. The genome sequence of passion fruit has been published recently [28,29] which enables us to study the function and characteristics of passion fruit genes.

*SBP* genes play a critical role in plant growth and development. However, genome-wide characterization and functional analysis of SBP transcription factors in passion fruit have not yet been carried out. In this study, a total of 14 *SBP* genes were identified in the passion fruit genome and divided into eight groups. The gene structure, conserved motifs, synteny analysis, and expression analyses were presented. In addition, the expression patterns of *PeSBP* genes in various stress responses were studied. The systematic analysis provided a foundation for further functional characterization of *SBP* genes in passion fruit.

## 2. Results

### 2.1. Identification and Characterization of SBP Genes in Passion Fruit

**Figure 1:**
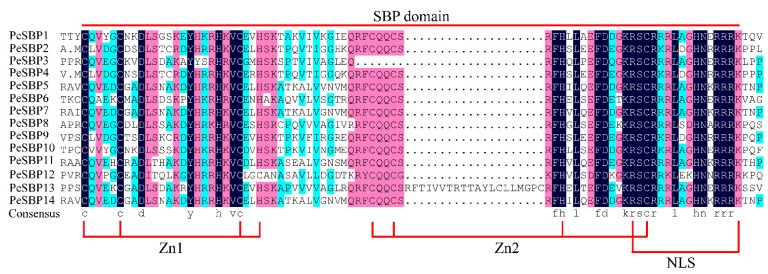
Multiple sequence alignment of SBP domains of PeSBP proteins. The darkblue, purple, and lightblue backgrounds indicate entire conservative residues, 75% conservative residues, and 50% conservative residues respectively.

To identify *PeSBP* genes in passion fruit, HMMER software and CDD online tools were used. A total of 14 *SBP* gene sequences were identified and named from *PeSBP1* to *PeSBP14* according to the location of the genes on the chromosomes (Supplementary Materials Table S1). Sequence alignment was performed with 14 SBP proteins in passion fruit to predict the SBP domain. The results showed that all PeSBP proteins had typical characteristics of the SBP domain, including two zinc domains (Zn1 and Zn2) and a nuclear localization signal (NLS) (Figure 1). With CDS ranging from 552 to 2943 bp, the full length of the 14 PeSBP proteins varied from 183 (PeSBP2) to 981 (PeSBP7) amino acid residues, with the molecular mass ranging from 20.57 (PeSBP4) to 108.28 (PeSBP5) kDa and isoelectric points ranging from 5.93 (PeSBP5) to 9.64 (PeSBP4). The total average hydrophilicity (GRAVY) values were all negative, indicating that all PeSBPs are hydrophilic. The subcellular localization results showed that thirteen of the fourteen PeSBP proteins were located in the nucleus (Supplementary Materials Table S1), while the PeSBP12 was located in the chloroplast. That may be because the nuclear localization sequence of chloroplast localization proteins will be removed after the protein enters the chloroplast. So, the protein will not move to the nucleus from the chloroplast. Furthermore, we identified the orthologous gene of *PeSBPs* in *Arabidopsis*. The orthologous gene and the function in *Arabidopsis* are shown in Supplementary Materials Table S1.

### 2.2. Phylogenetic Analysis of Passion Fruit SBP Gene Family

**Figure 2:**
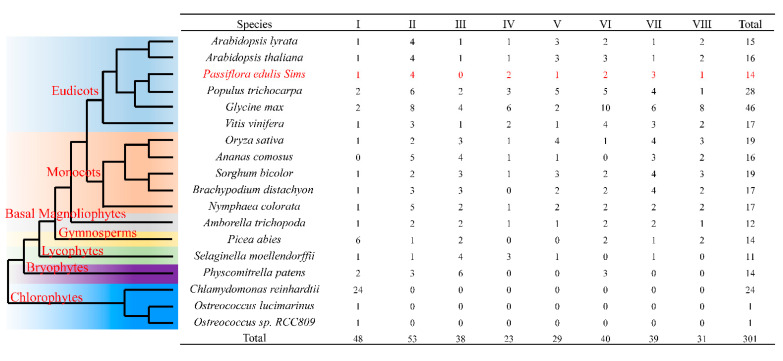
The evolutionary relationship of the SBP gene family of 18 species among seven lineages. Different green plant families are distinguished by different backgrounds.

**Figure 3:**
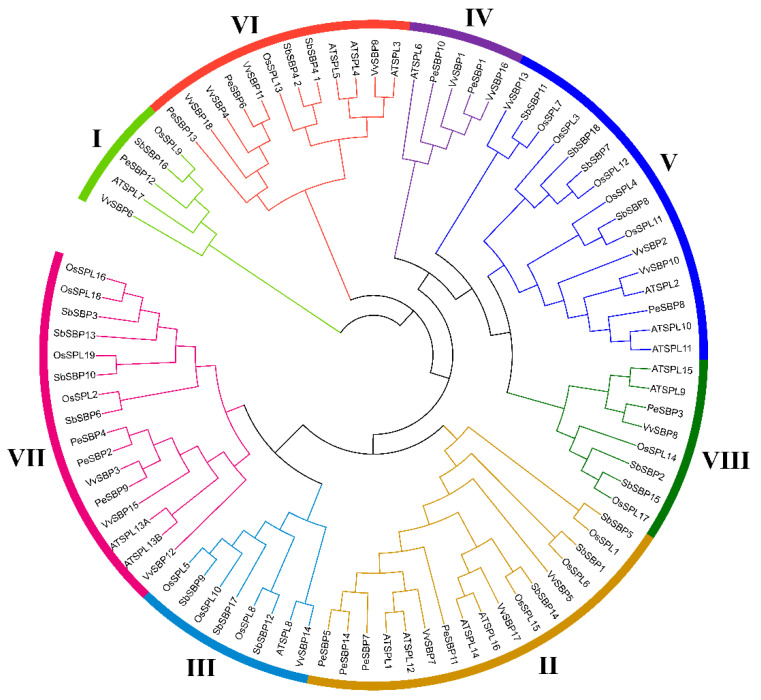
Phylogenetic tree of SBP family proteins among passion fruit, rice, *Arabidopsis*, grape, and sorghum. Pe, *Passiflora edulis* Sims; Os, *Oryza sativa*; AT, *Arabidopsis thaliana*; Vv, *Vitis vinifera*; and Sb, *Sorghum bicolor*.

To analyze the phylogeny of the *SBP* gene family, a total of 301 SBP homologs were selected from 18 representative plants from seven green plant families. Among these plants, the soybean had the maximum number of *SBP* genes, with 46 members. However, only one *SBP* gene in group I was found in *Ostrecocus lucimarinus* and *Ostrecocus* sp. *rcc809* (Figure 2). To further understand the phylogenetic relationship of the *PeSBP* gene family, a phylogenetic tree was constructed using SBP proteins of passion fruit, *Arabidopsis*, grape, sorghum, and rice. Among them, passion fruit, *Arabidopsis*, and grape belong to eudicots, while sorghum and rice belong to monocots. According to the unrooted phylogenetic tree, 14 *PeSBPs* could be divided into 8 groups (I to VIII). Four *PeSBPs* belonged to group II, while none belonged to group III. There was only one member of *PeSBP* in groups I, V, and VIII. The homology between the *PeSBPs* gene and the *VvSBPs* was relatively high, and the similarity between their protein sequences indicates that the *SBP* gene of these two species may have similar biological functions (Figure 3).

### 2.3. Gene Structure and Conserved Motif Analysis of SBP Genes in Passion Fruit

**Figure 4:**
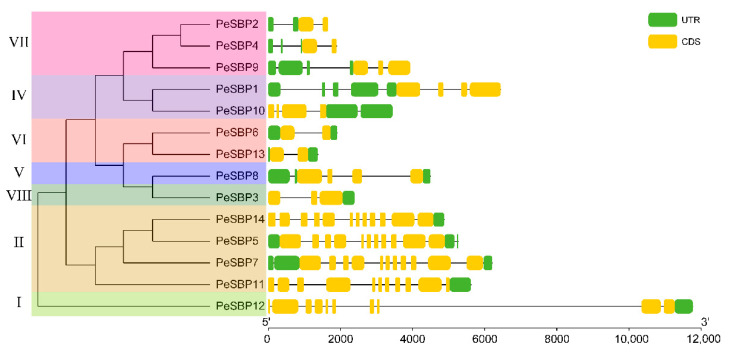
The exon–intron structure of *SBP* genes in passion fruit. The yellow round-corner rectangle represents CDS, the black shrunk line represents introns, and the green round-corner rectangle represents UTR.

The structure of each gene, especially the number and distribution of exon and intron, may be closely related to evolution. To better understand the diversity of *PeSBP* gene structure, the coding sequence of each gene was compared with its corresponding genomic sequence. The number of exons varied from 2 to 12. The number of introns in group II was the maximum, while all *PeSBP* genes in group VI only contained 1 intron, which was the lowest number. Group VII contained two to five introns, and group V contained 4 introns (Figure 4). In group II, *PeSBP5*, *PeSBP7*, *PeSBP 11*, and *PeSBP14* in the evolutionary tree may have been cleaved and polymerized during the evolutionary process. The results showed that the number of exons and introns was highly variable in different groups, while the number of exons was nearly consistent in the same group.

**Figure 5:**
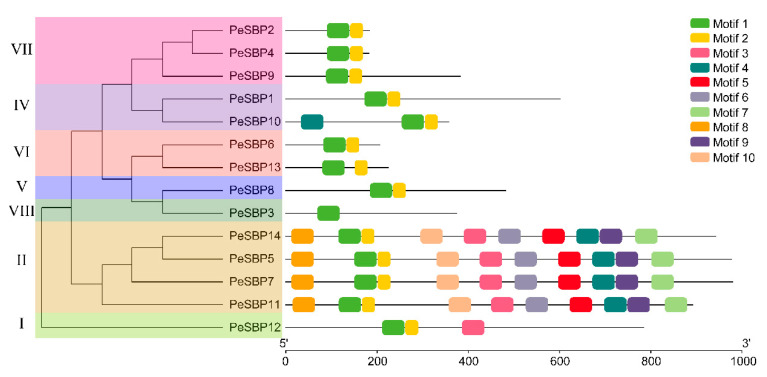
Distribution of the conserved motifs in passion fruit SBP proteins. Ten identified conserved motifs are marked in different colored boxes.

The number and types of conserved motifs contained in each protein sequence are different, which may reveal the different functions of each gene. A total of 10 motifs were identified in the PeSBP proteins and named motifs 1 to 10 (Supplementary Materials Figure S1). The results showed that all SBP members contained Motif 1 and Motif 2, suggesting that Motif 1 and Motif 2 constituted the conserved SBP domain (Supplementary Materials Table S2). PeSBP members in group II contained the maximum number of conservative motifs. PeSBP proteins clustered in the same group tended to have similar motif numbers. In contrast, some motifs were specific in some groups of PeSBP proteins—Motif 8, Motif 10, Motif 6, Motif 5, Motif 9, and Motif 7 were only found in group II, and Motif 4 was only found in PeSBP10, suggesting that some proteins may have specific biological functions under specific conditions (Figure 5). The majority within the same group exhibited similarity in motif compositions. Besides, the exon–intron structure of SBP proteins supported the phylogenetic analysis of SBP family genes, and the differences among the different groups indicate their diverse functions.

### 2.4. Synteny Analysis of PeSBP Genes

**Figure 6:**
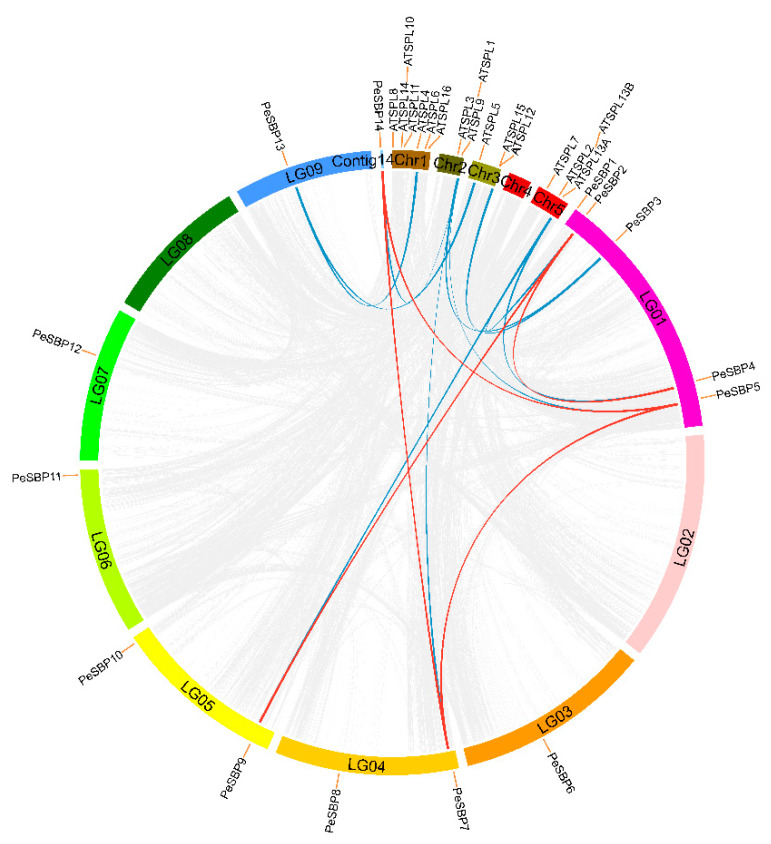
Synteny analysis of the *SBP* genes from passion fruit and *Arabidopsis*. The red lines indicate *SBP* gene pairs within passion fruit, and the blue lines indicate *SBP* gene pairs between passion fruit and *Arabidopsis*. Chr indicates the chromosome of *Arabidopsis*, and LG and contig indicate the chromosome of passion fruit.

The distribution analysis of *PeSBP* genes on the chromosomes indicated that 13 *PeSBP* genes map to seven chromosomes, except LG2 and LG8, while *PeSBP14* was located on P_eduliaContig 140022932.g. Since the passion fruit chromosome assembly and anchoring rate was only 96.07%, the PeSBP14 (P_ eduliaContig140022932.g) was not anchored to any chromosome but was assembled to Contig14. Among them, LG1 had the maximum number of *PeSBP* genes, including 5 *PeSBP* members. LG4 and LG5 chromosomes contain 2 *PeSBP* genes. The remaining four chromosomes had only 1 *PeSBP* gene (Figure 6). MCScanX software was used to analyze the collinearity of *SBP* genes between passion fruit and *Arabidopsis*. The blue line represents the collinearity of *SBP* genes between passion fruit and *Arabidopsis*, and the red line represents the synteny gene pairs of *SBP* genes between different chromosomes of passion fruit. A total of 16 pairs of collinear genes were identified, including 5 pairs of collinear genes within passion fruit and 11 pairs of synteny gene pairs between passion fruit and *Arabidopsis*. It can also be seen from the chromosome distribution diagram that there is still a collinearity relationship between *PeSBP14* and other genes on chromosomes. However, *PeSBP14* was located on the contig rather than the chromosome (Figure 6). It indicated that segmental duplication events might play a more important role in the evolution of the passion fruit *SBP* gene family. To further understand the evolutionary relationship of the *SBP* genes, we calculated Ka and Ks values to predict selection pressure for synteny gene pairs. Ka/Ks = 1 indicates neutral selection for homologous genes, Ka/Ks > 1 indicates that these genes evolved under positive selection, and Ka/Ks < 1 indicates that these genes had purification selection [30]. The results showed that the Ka/Ks values of all the *SBP* homogenous gene pairs within passion fruit and between passion fruit and *Arabidopsis* were less than 1, indicating that they may play a role in purification selection in the evolutionary process (Supplementary Materials Table S3).

### 2.5. Expression Profiles of PeSBP Genes in Different Tissues

Recent studies in various species have indicated that the *SBP* gene plays an important regulatory role in processes associated with plant flowering and inflorescence development [31]. To investigate the tissue-specific expression pattern of *PeSBP* genes in passion fruit flower development, we analyzed RNA-sequencing data from NGCB to determine the tissue-specific expression patterns of different passion fruit flower tissues (including the mixed flower, bract, sepal, petal, corona filament, stamen, stigma, and ovule at different development processes) and vegetative tissues (including the seed, root, and leaf).

**Figure 7:**
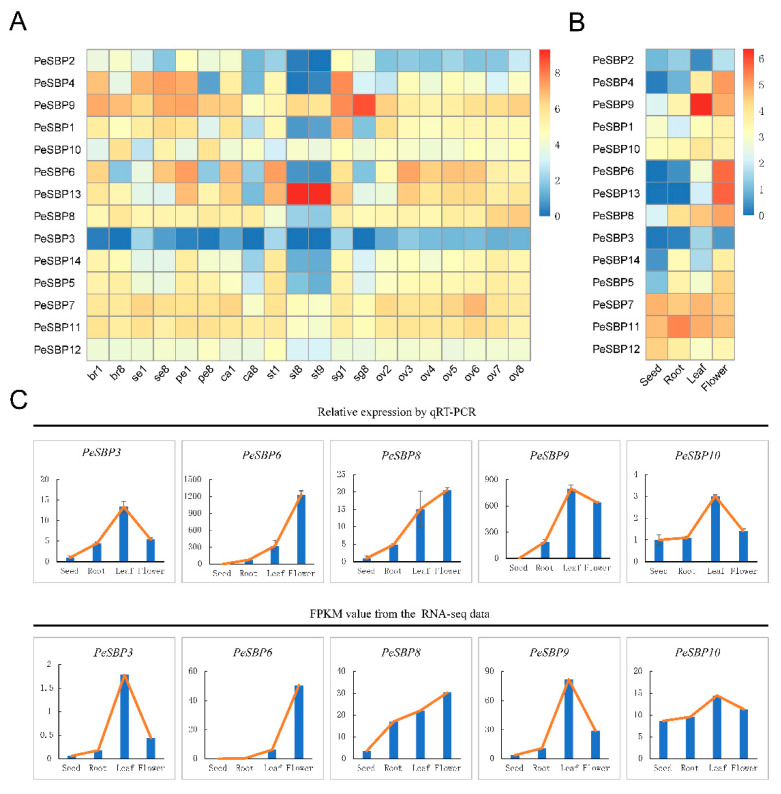
The expression pattern and qRT-PCR results of *SBP* genes in passion fruit. (**A**) Expression patterns of *SBP* genes in different tissues and different development stages based on the transcriptome data. br, bract; se, sepal; pe, petal; ca, corona filament; st, stamen; sg, stigma; and ov, ovule. The number represents different stages: 1 and 2 represent early stage, while 7 and 8 represent late stage. (**B**) Expression patterns of *SBP* genes in different tissues in the seed, root, leaf, and flower. The red color bar indicates high expression patterns, and the blue color bar indicates low expression. (**C**) Validation of 5 randomly selected *PeSBP* genes by qRT-PCR. The yellow line indicates the expression trend in four tissues.

According to their expression patterns, we found that the *PeSBP* genes showed a tissue-specific expression pattern. For instance, *PeSBP13* had high expression during pollen development, while *PeSBP9* had specifically high expression in the stigma, and *PeSBP4* had increased expression in the sepal and early stigma stages (Figure 7A, Supplementary Materials Table S4). The expression level of *PeSBP6*, *PeSBP8*, and *PeSBP13* genes were higher in flowers and leaves but lower in seeds and roots (Figure 7B, Supplementary Materials Table S4), suggesting that these three genes may play an important role in passion fruit flower and leaf development. The expression levels of *PeSBP7*, *PeSBP10*, *PeSBP11*, and *PeSBP12* were high in all tissues, indicating that they might be involved in various physiological processes of passion fruit flower development. Strangely, the expression levels of *PeSBP3* in all tissues were significantly lower than in other genes. These results suggest that diverse *PeSBPs* might be involved in the different floral tissues of passion fruit.

Furthermore, to validate the expression patterns of the *PeSBP* genes in the root, leaf, seed, and flower tissues, five *PeSBP* genes (*PeSBP3*, *PeSBP6*, *PeSBP8*, *PeSBP9*, and *PeSBP10*) were randomly selected to test their expression level in these four tissues by qRT-PCR. The qRT-PCR results were consistent with our RNA-sequencing results (Figure 7C), suggesting that all the *PeSBP* gene expression patterns in different tissues are reliable.

### 2.6. In Silico Promoter Analysis and Expression Profiles of PeSBP Genes in Response to Abiotic Stresses

**Figure 8:**
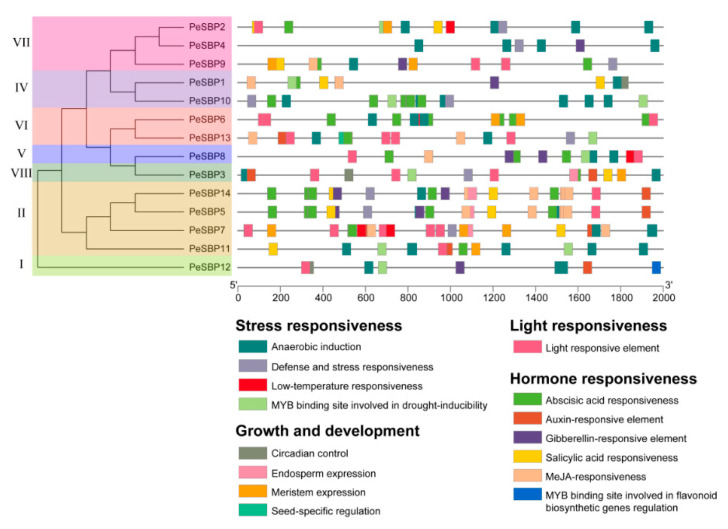
The distribution of *cis*-acting elements identified in the 2000-bp upstream promoter region of *PeSBP* genes.

To explore the mechanisms of *PeSBPs* in response to various stresses, we analyzed *cis*-elements of *PeSBPs* promoters. Two kb upstream genomic DNA sequences with transcriptional start site (ATG) from 14 *PeSBP* genes were analyzed. Four different classes of *cis*-elements were found in *PeSBPs* promoter regions, including stress responsiveness, light responsiveness, growth and development, and hormone responsiveness (Figure 8). The distribution of *cis*-elements in *PeSBP* genes is different, indicating that the expression of *PeSBPs* may vary in stressful, developmental, and hormonal contexts. Most *PeSBP* genes contained regulatory elements ABRE, ERE, CGTCA-motif, MBS, and W-box (Supplementary Materials Table S5). These results indicated that various types of *cis*-element in the same type of *PeSBP* genes might have different functions. ABRE is involved in abscisic acid responsiveness [32], while CGTCA-motif is involved in MeJA-responsiveness, and MBS (MYB binding site) is involved in drought inducibility. W-box is an important class of *cis*-element found in the promoter of plant defence response-related genes [33]. Gene ontology (GO) enrichment analysis of the PeSBPs was performed to understand their possible functions at the molecular level, i.e., molecular function (MF), biological process (BP), cellular component (CC). In MF class, *PeSBPs* enrichment in DNA binding, transcription regulator activity, nucleic acid binding, and so on (Supplementary Materials Figure S2). Five terms were identified as belonging to the CC class (Supplementary Materials Figure S2). Most promoters of *PeSBPs* genes were significantly enriched in the binding sites of transcription factors involved in the BP class in terms of regulation of nucleic acid-templated transcription, regulation of DNA-templated transcription, and regulation of gene expression (Supplementary Materials Figure S2). The information regarding the enrichment annotation results of GO terms for MF, CC, and BP is provided in Supplementary Materials Table S6. These results show that *SBP* gene family members may play an important role in different regulations.

**Figure 9:**
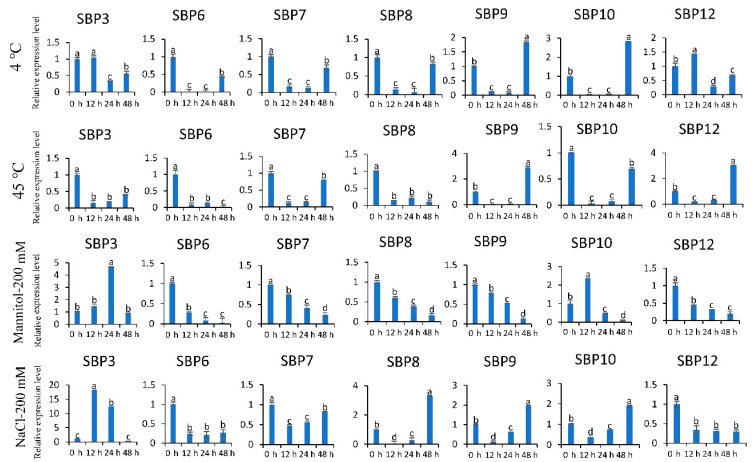
The relative expression levels of seven *SBP* genes in response to different stress treatments in passion fruit. Significant differences between samples labeled a, b, c, and d were determined by one-way ANOVA, *p* < 0.05.

To further confirm whether different abiotic stresses influence the expression of *PeSBP* genes, qRT-PCR experiments were performed to analyze the *SBP* gene expression patterns in response to different treatments, including cold, heat, and osmotic stress. As shown in Figure 9, seven *SBP* genes were selected for transcription-level detection. Under cold treatment, five genes (*PeSBP6/7/8/9/10*) had similar expression patterns that initially decreased and subsequently increased during the course of the treatment, while the relative expression level *PeSBP3/12* first increased and then decreased with the treatment time. During the heat treatment, three genes (*PeSBP3/6/8*) showed a continuous decrease with the treatment time, and the other four genes (*PeSBP7/9/10/12*) first decreased and then increased their expression with the treatment time.

The *cis*-elements in promoter regions are essential for regulating the gene expression of different stress response pathways. As shown above, we found some low-temperature responsiveness elements (LTR) in the promoter of *PeSBP6*, *PeSBP7*, and *PeSBP8* (Figure 8). Additionally, the qRT-PCR results showed that the LTR regulates the expression of *PeSBP6*, *PeSBP7,* and *PeSBP8* in response to temperature stress. Similarly, the MYB binding site involved in drought-inducibility was found in the *PeSBP3/8/10/12* promoter region. After osmotic stress, the transcription of *PeSBP8/12* continuously decreased with the treatment time, while the expression of *PeSBP3/10* first showed an increase and then decreased with the treatment time. It indicates that the MBS is important as it participates in regulating the expression of *PeSBP3/8/10/12* response to osmotic stress. After salt treatment, the expression level of *PeSBP8/9/10* increased gradually, while the expression of *PeSBP6/7/12* was repressed, and the expression of *PeSBP3* increased within 24 h and then decreased 48 h after treatment. Altogether, different *SBP* genes display differential expression patterns after stress treatment, indicating that these genes might function in response to abiotic stresses.

## 3. Discussion

The *SBP* gene family is a plant-specific transcription factor family widely found in green plants, and it plays a crucial role in plant growth and development, physiological and biochemical activities, and environmental response [34]. Passion fruit is a famous tropical and subtropical crop which has extensive economic value [35]. However, only a few studies on the *SBP* gene family in passion fruit exist. In this study, a total of 14 *SBP* genes were identified from the passion fruit genome via the bioinformatics method, which was similar in number to other eudicot plants, like *Arabidopsis thaliana* (16), *Arabidopsis lyrata* (15), and *Vitis vinifera* (17), but fewer in number than *Glycine max* (46) and *Populus trichocarpa* (28), suggesting that *SBP* genes in different eudicot species underwent different gene duplication events [36]. There was only one *SBP* gene in *Ostrecocus lucimarinus* and *Ostrecocus* sp. *rcc809* in group I, leading us to speculate that the *SBP* gene of group I might be their ancestor. We found that the number of *SBP* genes increased in terrestrial plants, and gene expansion was intensified strongly in angiosperms, which may reflect the flowering plants’ effectiveness in adapting to the different unstable environmental conditions [37]. According to phylogenetic tree analysis, the *SBP* gene family of passion fruit could be divided into eight groups. This result is consistent with previous *SBP* gene studies in other plants [30]. No *PeSBP* gene belonging to group III was identified. Whether these genes were lost in the evolution of passion fruit remains to be explored. Most *PeSBP* genes were clustered closely with the SBP family genes of grape and *Arabidopsis* but were far away from the *SBP* genes of rice. This finding is consistent with the fact that passion fruit, grape, and *Arabidopsis* are eudicot plants. Compared with the lineage that led to the formation of monocots, it is closer to the divergence in evolution from a common ancestor.

The isoelectric points of PeSBPs were between 5.93–9.64, indicating that most PeSBP proteins are rich in basic amino acids, and they may play a role in an acidic subcellular environment. Genes clustered in an evolutionary clade may have similar genetic structures and functions and often have closer relationships [38]. The gene structure of *PeSBPs* was relatively simple, and the number of exons ranged from 2 to 12. Most *PeSBP* genes in the same group shared a similar exon–intron structure. The exon –intron structure varied significantly among different groups, suggesting that the gene structure is closely related to the function. Motif identification is particularly important to explore new members of the gene family. Conserved motif analysis results showed that all PeSBP proteins contained motif 1 and motif 2, suggesting that motif 1 and motif 2 constitute the SBP domain. There were some differences in the number of motifs among different groups, with group II possessing ten motifs and groups V, VI, IV, and VII only having two motifs (motif 1 and motif 2). In general, gene structure and conserved motif analysis can support the grouping of evolutionary trees and are associated with specific biological functions [39].

Gene duplication events (segmental and tandem) are the major driving forces for finding novel genes and gene family expansion which can support the adaptation of organisms to different complex environments [40]. Collinearity analysis mainly evaluates the remaining and lost duplication genes through homology comparison. It can provide an important basis for studying the evolutionary history of genes by comparing the homologous gene sequences of different plant genomes [41]. Colinear analysis revealed the evolutionary relationship between homologous gene pairs within passion fruit and between passion fruit and *Arabidopsis*. A total of 5 segmental duplication events of collinear genes were found within passion fruit, and 11 duplication synteny gene pairs were found between passion fruit and *Arabidopsis,* indicating segmental duplication events produced the diversity of *PeSBPs*. The Ka/Ks values of SBP collinearity gene pairs in all identified homologous gene pairs were less than 1, suggesting that SBP collinearity gene pairs underwent purification selection in the evolution process with no functional change.

Plant SBP transcription factors play an important role in plant development, including flower development [42], fruit development [36], plant hormone signal transduction [43], and vegetative to reproductive phase transition [44,45]. An analysis of *cis*-acting elements of the *PeSBP* gene promoters showed that the promoters of *SBP* genes contained multiple *cis*-acting regulatory elements, which regulate multiple different growth and development and stress tolerance processes. The regulatory elements of different *SBP* genes in passion fruit were different, which might be closely related to the functional diversity of *SBP* genes. In *Arabidopsis*, *AtSPL6* is a positive regulator of defence gene expression [46]. The phylogenetic tree results showed that *AtSPL6* are orthologous to *PeSBP1* and *PeSBP10* in group IV. Interestingly, the *cis*-element analysis showed that *PeSBP1* and *PeSBP10* contain multiple defence and stress-responsive elements, such as plant defence response, defence and stress responsiveness, and the MYB binding site involved in drought-inducibility. We speculate that *PeSBP1* and *PeSBP10* might also be positive regulators of defence gene expression in passion fruit. However, the role of *cis*-elements in the *PeSBP* gene promoter regions in stress response requires further investigation. To further understand the possible role of *PeSBP* genes, we examined the expression levels of 14 *SBP* genes in different tissues of passion fruit. The results showed tissue-specific *SBP* gene expression during the growth and development of passion fruit, and the expression levels of the same gene were different in different tissues. In addition, most *PeSBP* genes were highly expressed in leaf and flower tissues. These results were similar to *SBP* genes of other species and were highly expressed in the shoot development, apical buds inflorescences, and flower buds [47]. For instance, *PeSBP8* in group V was orthologous to *AtSPL2*, *AtSPL10*, and *AtSPL11* [48] and expressed highly in the passion fruit leaves, suggesting that *PeSBP8* plays an important role in the regulation of leaf development. The mutant of *AtSPL1* and *AtSPL12* inflorescences displayed hypersensitivity to heat stress, whereas the overexpression of *AtSPL1* or *AtSPL1*2 enhanced the thermotolerance in *Arabidopsis* [49]. *PeSBP5*, *PeSBP7*, and *PeSBP14* were orthologous to *AtSPL1* and *AtSPL12*, expressed highly in the passion fruit ovule and flower stages. The *cis*-element analysis also showed that *PeSBP5*, *PeSBP7,* and *PeSBP14* promoters contained many growth, development, and stress response related elements, such as binding sites for transcription factors involved in the regulation of endosperm expression, meristem expression, LTR, and defence and stress responsiveness (Figure 8, Supplementary Materials Table S5). These results may improve the cognition of *PeSBPs* and furnish potential clues for further studies of SBP family genes in passion fruit.

## 4. Materials and Methods

### 4.1. Plant Materials

Two-month-old plants were used for different treatments. Passion fruit cultivar Tainong No. 1 seeds were germinated in the greenhouse at 30 ± 1 °C, with a relative humidity of 70%, and a 16-h light/8-h dark photoperiod. At two true leaves stages, the seedlings were transferred to the growth chamber and grown for an additional two months. The materials were provided by the passion fruit breeding group at Fujian Agriculture and Forestry University.

### 4.2. Identification and Characterization Analysis of SBP Genes in Passion Fruit

First, SBP protein sequences were downloaded from National Genomics Data Center (NGDC) (accession number GWHAZTM00000000). A hidden Markov model (HMM) map of the *SBP* gene family (PF03110) was obtained from the *Pfam* database [50]. *SBP* genes were identified from the whole genome of passion fruit using HMMER (v3.2.1, http://hmmer.org/ (accessed on 22 November 2021)) software and the BLAST program (v2.12.0, ftp://ftp.ncbi.nlm.nih.gov/blast/executables/blast+/2.12.0/ (accessed on 22 November 2021)), and the candidate genes were further confirmed by containing SBP domain using CDD (https://www.ncbi.nlm.nih.gov/cdd/, (accessed on 22 November 2021)) and SMART server (http://smart.embl.de/, (accessed on 22 November 2021)) online tools, and sequences without complete SBP domains were deleted. Finally, DNAMAN software (v8, LynnonBiosoft, USA) was used to perform multiple sequence alignments on all candidate genes to ensure that they contained the SBP domain. Meanwhile, ExPASy Server (https://web.expasy.org/protparam/ (accessed on 9 December 2021)) [51] was used to calculate the isoelectric point (pI) and the molecular weight (MW), and the length of the amino acids, introns, exons, and open reading frames (ORF) were also calculated. Subcellular localization of SBP protein was predicted using BaCelLo (Balanced Subcellular Localization Predictor) software (http://gpcr2.biocomp.unibo.it/bacello/index.htm, (accessed on 9 December 2021)) [52].

### 4.3. Sequence Alignments and Phylogenetic Analysis

MUSCLE software was used to compare the amino acid sequences of the *SBP* gene family. To study the evolutionary relationship of *SBP* genes, a phylogenetic tree was constructed among passion fruit, *Arabidopsis*, grape, sorghum, and rice using MEGA (v6.0, Tokyo Metropolitan University, Hachioji, Tokyo, Japan) with the maximum likelihood method. The following parameters were used: JTT model, pairwise deletion, and the bootstrap test, which were replicated 1000 times. The phylogenetic tree was imported into the Interactive tree of life (iTOL, https://itol.embl.de/ (accessed on 12 December 2021)) [53].

### 4.4. Gene Structure and Conserved Motif Analysis

The Gene Structure Display Server (GSDS, http://gsds.gao-lab.org/https://itol.embl.de/ (accessed on 15 December 2021)) was used to determine the exon–intron structure of passion fruit *SBP* genes based on their genome DNA sequence and CDS sequence [54]. Then, the protein sequence of the passion fruit *SBP* gene family was submitted to the MEME online tool (https://meme-suite.org/meme https://itol.embl.de/ (accessed on 15 December 2021)) [55] for conserved motif prediction, and the number of motif discovery was set to 10 with default parameters. TBtools was used to visualize the prediction motif results of the *SBP* gene family in passion fruit [56].

### 4.5. Chromosomal Locations and Synteny Analysis

The 14 passion fruit SBP family genes were located on the chromosomes using the information annotated in the passion fruit genome. The physical location of *PeSBPs* on the chromosomes was visualized using Circos software (v0.69, http://www.circos.ca/ (accessed on 19 December 2021)). A synteny analysis of the *SBP* gene family in the passion fruit genome was conducted using MCScanX software (http://chibba.pgml.uga.edu/mcscan2 (accessed on 19 December 2021)). Circos software also was used to visually map synteny genes. The easy_Kaks calculation program (https://github.com/tangerzhang/FAFU-cgb/tree/master/easy_KaKs (accessed on 19 December 2021)) was used to calculate the non-synonymous (Ka), synonymous (Ks), and Ka/Ks substitution ratios of the homologous gene pairs of passion fruit and *Arabidopsis* to estimate the selection and substitution rates.

### 4.6. Cis-Elements Analysis of PeSBPs Promoters

The promoter sequence of *PeSBP* genes was 2 kb upstream of the transcriptional start site (ATG). The *cis-*elements of the promoter of the *PeSBP* genes were predicted using the Plant Cis-Acting Regulatory Element (PlantCARE, http://bioinformatics.psb.ugent.be/webtools/plantcare/html/ (accessed on 19 December 2021)) [57].

### 4.7. Expression Patterns Analysis

The RNA-sequencing data of the different floral tissues, including bract, sepal, corona filament, petal, stamen, stigma, and ovule, were obtained from China National GenBank (CNGB) (accession number CNP0002747), and the RNA-sequencing results of the root, flower, leaf, and seed tissues were downloaded from (https://ngdc.cncb.ac.cn/gsa/browse/CRA003773 (accessed on 19 December 2021)). The heatmap was then constructed using the pheatmap package of R software.

### 4.8. RNA Extraction and Quantitative Real-Time PCR

High-quality RNA was extracted using plant total RNA extraction kits (Omega Bio-Tek, Shanghai, China) and then reverse-transcribed into cDNA using a reverse transcription kit (TaKaRa, Beijing, China). A SYBR Premix Ex Taq kit (TaKaRa, Beijing, China) was used for qRT-PCR to verify the expression levels of each sample. Three replicates were designed for each sample, and the *EF1a* gene was used as a reference to calculate the relative expression levels of *SBP* genes in various tissues. Primers were designed to amplify sequences in PrimerQuest tools (Supplementary Materials Table S7).

### 4.9. PeSBPs Response to the Abiotic Stresses

Two-month-old passion fruit plants (variety in Tainong No. 1) in soil were analyzed in response to the abiotic stress treatment. For cold (4 °C) and heat (45 °C) stress, seedlings in soil were transferred to the incubator at 16/8 h, day/night. For salt and osmotic stress treatment, the passion fruit plants were first grown in the liquid ½MS medium for 7 days and then transferred to fresh liquid ½MS medium with 200 mM NaCl or 200 mM mannitol for stress treatment. Leaves were collected from at least three independent plants at 12 h, 24 h, and 48 h after treatment, and the controls were not subjected to any stress treatments. Three replicates were performed for each treatment. The collected samples were immediately stored in liquid nitrogen before total RNA extraction. The expression profiles of *PeSBPs* were detected using qRT-PCR.

### 4.10. Gene Ontology (GO) Enrichment Analysis

GO enrichment analysis was carried out using AgriGO (http://bioinfo.cau.edu.cn/agriGO/ (accessed on 1 November 2022)), and the result was visualized using R software (https://cran.r-project.org/web/packages/pheatmap/index.html (accessed on 1 November 2022)).

## 5. Conclusions

In the present study, a total of 14 *SBP* genes were identified in passion fruit. Based on the phylogenetic, gene structure, and conserved motifs analysis, all *PeSBPs* were classified into eight groups. The major gene expansion of the *PeSBP* gene family was segmental duplication and under negative selection. *Cis*-element analyses showed that *PeSBPs* responded to different stresses and plant hormones. In addition, the study of expression patterns under various stress types suggested that *PeSBPs* function across multiple stress responses. Taken together, our comprehensive analyses are helpful in selecting candidate *SBP* genes to further understand the classification and function of the *SBP* gene family in passion fruit.

## Data Availability

Not applicable.

## Supplementary Materials

The supporting information can be downloaded at: https://www.mdpi.com/article/10.3390/ijms232214153/s1.

## References

[1] Amoutzias G.D., Veron A.S., Weiner J., Robinson-Rechavi M., Bornberg-Bauer E., Oliver S.G., Robertson D.L. (2007). One billion years of bZIP transcription factor evolution: Conservation and change in dimerization and DNA-binding site specificity. Mol. Biol. Evol..

[2] Du H., Yang S.S., Liang Z., Feng B.R., Liu L., Huang Y.B., Tang Y.X. (2012). Genome-wide analysis of the MYB transcription factor superfamily in soybean. BMC Plant Biol..

[3] Xiong Y.Q., Liu T.Y., Tian C.G., Sun S.H., Li J.Y., Chen M.S. (2005). Transcription factors in rice: A genome-wide comparative analysis between monocots and eudicots. Plant Mol. Biol..

[4] Guo A.Y., He K., Liu D., Bai S.N., Gu X.C., Wei L.P., Luo J.C. (2005). DATF: A database of Arabidopsis transcription factors. Bioinformatics.

[5] Guo A.Y., Chen X., Gao G., Zhang H., Zhu Q.H., Liu X.C., Zhong Y.F., Gu X.C., He K., Luo J.C. (2008). PlantTFDB: A comprehensive plant transcription factor database. Nucleic Acids Res..

[6] Yang Z.F., Wang X.F., Gu S.L., Hu Z.Q., Xu H., Xu C.W. (2008). Comparative study of SBP-box gene family in Arabidopsis and rice. Gene.

[7] Yamasaki K., Kigawa T., Inoue M., Tateno M., Yamasaki T., Yabuki T., Aoki M., Seki E., Matsuda T., Nunokawa E. (2004). A novel zinc-binding motif revealed by solution structures of DNA-binding domains of Arabidopsis SBP-family transcription factors. J. Mol. Biol..

[8] Hou H.M., Li J., Gao M., Singer S.D., Wang H., Mao L.Y., Fei Z.J., Wang X.P. (2013). Genomic Organization, Phylogenetic Comparison and Differential Expression of the SBP-Box Family Genes in Grape. PLoS ONE.

[9] Klein J., Saedler H., Huijser P.J.M.-M., Genetics G., Klein J., Saedler H., Huijser P. (1995). A new family of DNA binding proteins includes putative transcriptional regulators of the *Antirrhinum majus* floral meristem identity gene SQUAMOSA. Mol. Gen. Genet..

[10] Cardon G.H., Hohmann S., Nettesheim K., Saedler H., Huijser P. (1997). Functional analysis of the *Arabidopsis thaliana* SBP-box gene SPL3: A novel gene involved in the floral transition. Plant J..

[11] Tripathi R.K., Goel R., Kumari S., Dahuja A. (2017). Genomic organization, phylogenetic comparison, and expression profiles of the SPL family genes and their regulation in soybean. Dev. Genes Evol..

[12] Chang J.Z., Yan F.X., Qiao L.Y., Zheng J., Zhang F.Y., Liu Q.S. (2016). Genome-wide identification and expression analysis of SBP-box gene family in *Sorghum bicolor* L. Yi Chuan.

[13] Li C., Lu S. (2014). Molecular characterization of the SPL gene family in *Populus trichocarpa*. BMC Plant Biol..

[14] Zhang H.X., Jin J.H., He Y.M., Lu B.Y., Li D.W., Chai W.G., Khan A., Gong Z.H. (2016). Genome-Wide Identification and Analysis of the SBP-Box Family Genes under *Phytophthora capsici* Stress in Pepper (*Capsicum annuum* L.). Front. Plant Sci..

[15] Hultquist J.F., Dorweiler J.E. (2008). Feminized tassels of maize mop1 and ts1 mutants exhibit altered levels of miR156 and specific SBP-box genes. Planta.

[16] Salinas M., Xing S., Hohmann S., Berndtgen R., Huijser P. (2012). Genomic organization, phylogenetic comparison and differential expression of the SBP-box family of transcription factors in tomato. Planta.

[17] Lannenpaa M., Janonen I., Holtta-Vuori M., Gardemeister M., Porali I., Sopanen T. (2004). A new SBP-box gene BpSPL1 in silver birch (*Betula pendula*). Physiol. Plant..

[18] Unte U.S., Sorensen A.-M., Pesaresi P., Gandikota M., Leister D., Saedler H., Huijser P. (2003). SPL8, an SBP-Box Gene That Affects Pollen Sac Development in Arabidopsis. Plant Cell.

[19] Zhang B., Xu W.N., Liu X., Mao X.G., Li A., Wang J.Y., Chang X.P., Zhang X.Y., Jing R.L. (2017). Functional Conservation and Divergence among Homoeologs of TaSPL20 and TaSPL21, Two SBP-Box Genes Governing Yield-Related Traits in Hexaploid Wheat. Plant Physiol..

[20] Cui L., Zheng F.Y., Wang J.F., Zhang C.L., Xiao F.M., Ye J., Li C.X., Ye Z.B., Zhang J.H. (2020). miR156a-targeted SBP-Box transcription factor SlSPL13 regulates inflorescence morphogenesis by directly activating SFT in tomato. Plant Biotechnol. J..

[21] Luo L., Li W.Q., Miura K., Ashikari M., Kyozuka J. (2012). Control of Tiller Growth of Rice by OsSPL14 and Strigolactones, Which Work in Two Independent Pathways. Plant Cell Physiol..

[22] Wang S.K., Wu K., Yuan Q.B., Liu X.Y., Liu Z.B., Lin X.Y., Zeng R.Z., Zhu H.T., Dong G.J., Qian Q. (2012). Control of grain size, shape and quality by OsSPL16 in rice. Nat. Genet..

[23] Ning K., Chen S., Huang H.J., Jiang J., Yuan H.M., Li H.Y. (2017). Molecular characterization and expression analysis of the SPL gene family with BpSPL9 transgenic lines found to confer tolerance to abiotic stress in Betula platyphylla Suk. Plant Cell Tissue Organ Cult..

[24] Ortiz D.C., Bohorquez A., Duque M.C., Tohme J., Cuellar D., Vasquez T.M. (2012). Evaluating purple passion fruit (*Passiflora edulis* Sims f. *edulis*) genetic variability in individuals from commercial plantations in Colombia. Genet. Resour. Crop. Evol..

[25] Araya S., Martins A.M., Junqueira N.T.V., Costa A.M., Faleiroa F.G., Ferreira M.E. (2017). Microsatellite marker development by partial sequencing of the sour passion fruit genome (*Passiflora edulis* Sims). BMC Genom..

[26] Williams N. (2003). Passion flowers. Curr. Biol..

[27] Santos E.A., Souza M.M., Abreu P.P., da Conceicao L.D.H.C.S., Araujo I.S., Viana A.P., de Almeida A.A.F., Freitas J.C.D. (2012). Confirmation and characterization of interspecific hybrids of *Passiflora* L. (Passifloraceae) for ornamental use. Euphytica.

[28] Ma D.N., Dong S.S., Zhang S.C., Wei X.Q., Xie Q.J., Ding Q.S., Xia R., Zhang X.T. (2021). Chromosome-level reference genome assembly provides insights into aroma biosynthesis in passion fruit (*Passiflora edulis*). Mol. Ecol. Resour..

[29] Xia Z.Q., Huang D.M., Zhang S.K., Wang W.Q., Ma F.N., Wu B., Xu Y., Xu B.Q., Chen D., Zou M.L. (2021). Chromosome-scale genome assembly provides insights into the evolution and flavor synthesis of passion fruit (*Passiflora edulis* Sims). Hortic Res..

[30] Liu Y.H., Aslam M., Yao L.A., Zhang M., Wang L.L., Chen H.H., Huang Y.M., Qin Y., Niu X.P. (2021). Genomic analysis of SBP gene family in *Saccharum spontaneum* reveals their association with vegetative and reproductive development. BMC Genom..

[31] Li L., Shi F., Wang Y., Yu X., He G.J.P.S. (2020). TaSPL13 regulates inflorescence architecture and development in transgenic wheat (*Triticum aestivum* L.). Plant Sci..

[32] Fujita Y., Fujita M., Satoh R., Maruyama K., Parvez M.M., Seki M., Hiratsu K., Ohme-Takagi M., Shinozaki K., Yamaguchi-Shinozaki K. (2005). AREB1 is a transcription activator of novel ABRE-dependent ABA signaling that enhances drought stress tolerance in Arabidopsis. Plant Cell.

[33] Yamaguchi-Shinozaki K., Shinozaki K. (2006). Transcriptional regulatory networks in cellular responses and tolerance to dehydration and cold stresses. Annu. Rev. Plant Biol..

[34] Xu M.L., Hu T.Q., Zhao J.F., Park M.Y., Earley K.W., Wu G., Yang L., Poethig R.S. (2016). Developmental Functions of miR156-Regulated SQUAMOSA PROMOTER BINDING PROTEIN-LIKE (SPL) Genes in *Arabidopsis thaliana*. PLoS Genet..

[35] Cerqueira-Silva C.B.M., Jesus O.N., Santos E.S.L., Correa R.X., Souza A.P. (2014). Genetic Breeding and Diversity of the Genus Passiflora: Progress and Perspectives in Molecular and Genetic Studies. Int. J. Mol. Sci..

[36] Liu M.Y., Sun W.J., Ma Z.T., Huang L., Wu Q., Tang Z.Z., Bu T.L., Li C.L., Chen H. (2019). Genome-wide identification of the SPL gene family in Tartary Buckwheat (*Fagopyrum tataricum*) and expression analysis during fruit development stages. BMC Plant Biol..

[37] Corrêa L., Riaño-Pachón D., Schrago C.G., Santos R., Mueller-Roeber B., Vincentz M.J.P.O. (2008). The Role of bZIP Transcription Factors in Green Plant Evolution: Adaptive Features Emerging from Four Founder Genes. PLoS ONE.

[38] Xie K., Wu C., Xiong L. (2006). Genomic Organization, Differential Expression, and Interaction of SQUAMOSA Promoter-Binding-Like Transcription Factors and microRNA156 in Rice. Plant Physiol..

[39] Liu Y., Chai M., Zhang M., He Q., Qin Y.J.I.J.o.G. (2020). Genome-Wide Analysis, Characterization, and Expression Profile of the Basic Leucine Zipper Transcription Factor Family in Pineapple. J. Genom..

[40] Du H., Feng B.R., Yang S.S., Huang Y.B., Tang Y.X. (2012). The R2R3-MYB Transcription Factor Gene Family in Maize. PLoS ONE.

[41] Koonin E.V. (2005). Orthologs, paralogs, and evolutionary genomics. Annu. Rev. Genet..

[42] Gandikota M., Birkenbihl R.P., Hohmann S., Cardon G.H., Saedler H., Huijser P. (2007). The miRNA156/157 recognition element in the 3′ UTR of the Arabidopsis SBP box gene SPL3 prevents early flowering by translational inhibition in seedlings. Plant J..

[43] Zhang Y., Schwarz S., Saedler H., Huijser P. (2007). SPL8, a local regulator in a subset of gibberellin-mediated developmental processes in Arabidopsis. Plant Mol. Biol..

[44] Jung J.H., Seo P.J., Kang S.K., Park C.M. (2011). miR172 signals are incorporated into the miR156 signaling pathway at the SPL3/4/5 genes in Arabidopsis developmental transitions. Plant Mol. Biol..

[45] Yu S., Lian H., Wang J.W. (2015). Plant developmental transitions: The role of microRNAs and sugars. Curr. Opin. Plant Biol..

[46] Padmanabhan M.S., Ma S., Burch-Smith T.M., Czymmek K., Huijser P., Dinesh-Kumar S.P. (2013). Novel positive regulatory role for the SPL6 transcription factor in the N TIR-NB-LRR receptor-mediated plant innate immunity. PLoS Pathog..

[47] Wu G., Poethig R.S. (2006). Temporal regulation of shoot development in *Arabidopsis thaliana* by miR156 and its target SPL3. Development.

[48] Shikata M., Koyama T., Mitsuda N., Ohme-Takagi M. (2009). Arabidopsis SBP-box genes SPL10, SPL11 and SPL2 control morphological change in association with shoot maturation in the reproductive phase. Plant Cell Physiol..

[49] Chao L.M., Liu Y.Q., Chen D.Y., Xue X.Y., Mao Y.B., Chen X.Y. (2017). Arabidopsis Transcription Factors SPL1 and SPL12 Confer Plant Thermotolerance at Reproductive Stage. Mol. Plant.

[50] El-Gebali S., Mistry J., Bateman A., Eddy S.R., Luciani A., Potter S.C., Qureshi M., Richardson L.J., Salazar G.A., Smart A. (2019). The Pfam protein families database in 2019. Nucleic Acids Res..

[51] Artimo P., Jonnalagedda M., Arnold K., Baratin D., Csardi G., de Castro E., Duvaud S., Flegel V., Fortier A., Gasteiger E. (2012). ExPASy: SIB bioinformatics resource portal. Nucleic Acids Res..

[52] Pierleoni A., Martelli P.L., Fariselli P., Casadio R. (2006). BaCelLo: A balanced subcellular localization predictor. Bioinformatics.

[53] Letunic I., Bork P. (2019). Interactive Tree Of Life (iTOL) v4: Recent updates and new developments. Nucleic Acids Res..

[54] Hu B., Jin J., Guo A.Y., Zhang H., Luo J., Gao G. (2015). GSDS 2.0: An upgraded gene feature visualization server. Bioinformatics.

[55] Bailey T.L., Boden M., Buske F.A., Frith M., Grant C.E., Clementi L., Ren J., Li W.W., Noble W.S. (2009). MEME SUITE: Tools for motif discovery and searching. Nucleic Acids Res..

[56] Chen C., Chen H., Zhang Y., Thomas H.R., Frank M.H., He Y., Xia R. (2020). TBtools: An Integrative Toolkit Developed for Interactive Analyses of Big Biological Data. Mol. Plant.

[57] Lescot M., Dehais P., Thijs G., Marchal K., Moreau Y., Van de Peer Y., Rouze P., Rombauts S. (2002). PlantCARE, a database of plant cis-acting regulatory elements and a portal to tools for in silico analysis of promoter sequences. Nucleic Acids Res..

