# Supplementary material for: Genome-Wide Identification and Characterization of the SBP Gene Family in Passion Fruit (Passiflora edulis Sims)
Source: Int J Mol Sci. 2022 Nov 16;23(22):14153. doi: 10.3390/ijms232214153 (PMC9695787; doi:10.3390/ijms232214153)
Supplement: Supplementary file 1 [file ijms-23-14153-s001.zip › Supplementary legend.pdf]

Figure S1: 10 motifs of SBP proteins in passion fruit.; Figure S2: GO enrichment analysis of *PeSBP* genes.; Table S1: Primers used in the qRT-PCR analysis; Table S2: Information of identified *SBP* genes in passion fruit.; Table S3: The MEME motif sequences of PeSBPs.; Table S4: Synteny blocks of *SBP* genes between *Arabidopsis* and passion fruit.; Table S5: The expression profiles of *SBP* genes in passion fruit.; Table S6: *Cis*-elements in *PeSBP* promoters.; Table S7. The GO annotation and enrichment results of *PeSBPs*.
