# Supplementary figures and images for: Genome-Wide Identification and Characterization of the SBP Gene Family in Passion Fruit (Passiflora edulis Sims)
Source: Int J Mol Sci. 2022 Nov 16;23(22):14153. doi: 10.3390/ijms232214153 (PMC9695787; doi:10.3390/ijms232214153)

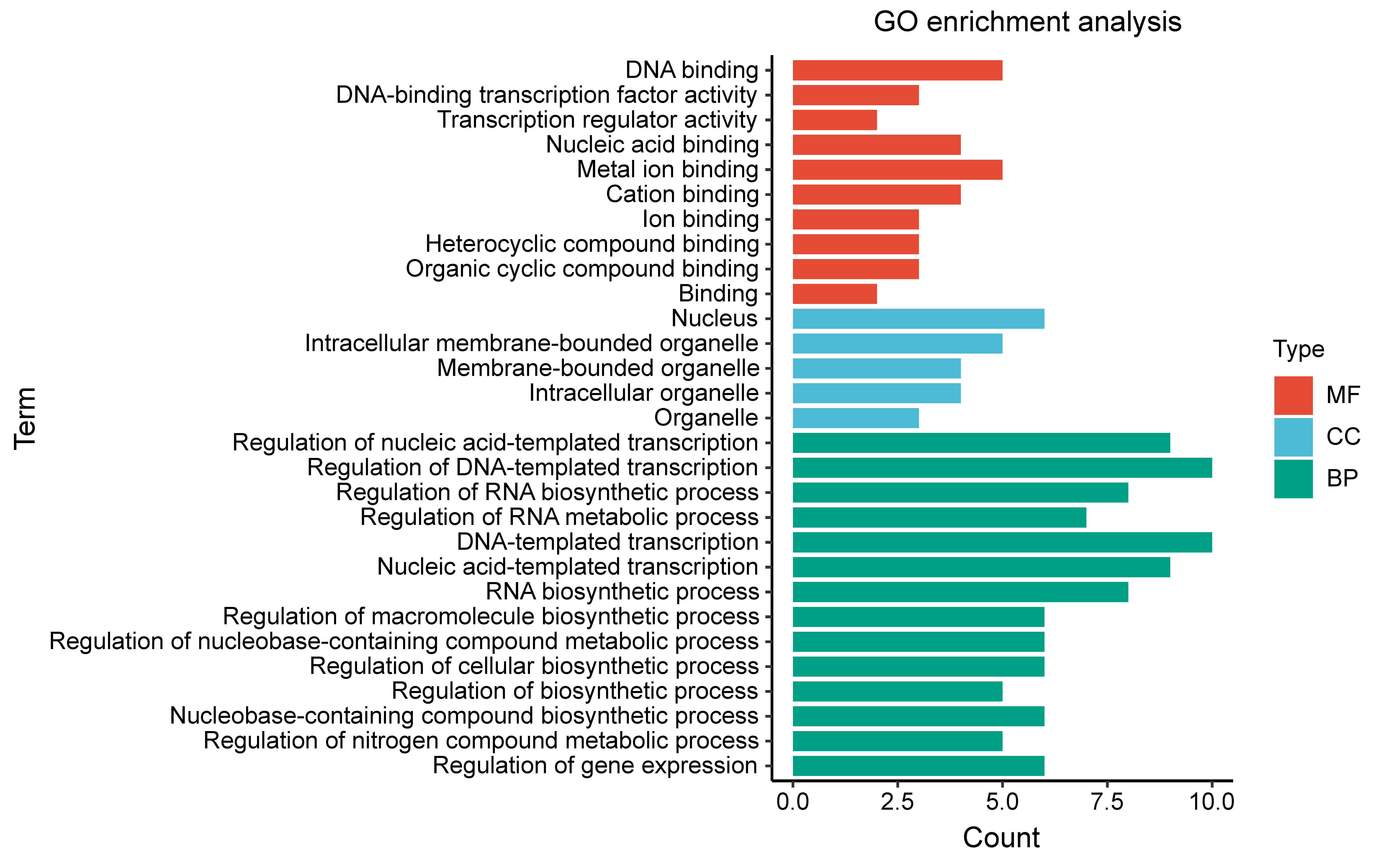

Supplement: Supplementary file 1 [file ijms-23-14153-s001.zip › Supplementary Materials Figure S2.tif]
